# Detection of smoking status from retinal images; a Convolutional Neural Network study

**DOI:** 10.1038/s41598-019-43670-0

**Published:** 2019-05-09

**Authors:** Ehsan Vaghefi, Song Yang, Sophie Hill, Gayl Humphrey, Natalie Walker, David Squirrell

**Affiliations:** 1 School of Optometry and Vision Sciences, University of Auckland, Auckland, New Zealand; 2 Auckland Bioengineering Institute, University of Auckland, Auckland, New Zealand; 3 Department of Ophthalmology, University of Auckland, Auckland, New Zealand; 4 National Institute for Health Innovation, University of Auckland, Auckland, New Zealand

**Keywords:** Eye diseases, Risk factors, Computational science

## Abstract

Cardiovascular diseases are directly linked to smoking habits, which has both physiological and anatomical effects on the systemic and retinal circulations, and these changes can be detected with fundus photographs. Here, we aimed to 1- design a Convolutional Neural Network (CNN), using retinal photographs, to differentiate between smokers and non-smokers; and 2- use the attention maps to better understand the physiological changes that occur in the retina in smokers. 165,104 retinal images were obtained from a diabetes screening programme, labelled with self-reported “smoking” or “non-smoking” status. The images were pre-processed in one of two ways, either “contrast-enhanced” or “skeletonized”. Experiments were run on an Intel Xeon Gold 6128 CPU @ 3.40 GHz with 16 GB of RAM memory and a NVIDIA GeForce TiTan V VOLTA 12 GB, for 20 epochs. The dataset was split 80/20 for training and testing sets, respectively. The overall validation outcomes for the contrast-enhanced model were accuracy 88.88%, specificity 93.87%. In contrast, the outcomes of the skeletonized model were accuracy 63.63%, specificity 65.60%. The “attention maps” that were generated of the contrast-enhanced model highlighted the retinal vasculature, perivascular region and the fovea most prominently. We trained a customized CNN to accurately determine smoking status. The retinal vasculature, the perivascular region and the fovea appear to be important predictive features in the determination of smoking status. Despite a high degree of accuracy, the sensitivity of our CNN was low. Further research is required to establish whether the frequency, duration, and dosage (quantity) of smoking would improve the sensitivity of the CNN.

## Introduction

Cardiovascular disease continues to be the leading cause of death globally^1^. One of the most important risk factors in the development of cardiovascular disease is cigarette smoking^2^. Smoking has both a physiological and anatomical effect on the systemic and retinal circulation^3^. Examining the retinal vasculature with fundus photography provides an exclusive opportunity to directly examine blood vessels non-invasively^4^. Fundus photos can thus be used to identify and monitor the progression of those eye diseases that have a systemic involvement.

Analysis of retinal images has revealed that there a number of biomarkers that are associated with increased cardiovascular risk. These include vessel tortuosity and bifurcation^5^ calibre^6–12^, microvascular changes^13,14^ and vascular fractal dimentions^15–17^. Smoking has been shown in a number of epidimological studies to influence the appearance of the retinal vasculature, resulting in wider retinal venular calibre^18,19^.

The technique of fundus photography has advanced and evolved rapidly over the last century^20^, and recently there has been a surge in the use of Deep Learning (DL) to analyse the retinal fundus photographs. DL is a subset of machine learning that involves providing a system with series of labelled examples of images of specific quality, so that the system can train itself to identify predictive features without explicit instructions. Convolutional Neural Networks (CNN) are a class of artificial neural networks, which use a variation of multilayer perceptrons and non-linear activation functions^21,22^.

Early studies used RIGA and SCES datasets and a custom CNN architecture for classification of optic-disk images to diagnose glaucoma^23^. This model was developed further to extract features and classify patients into those with or without glaucoma via a random forest classifier using a transfer learning of AlexNet^24^. Other modifications of AlexNet have been used in the detection of retinal lesions in diabetic retinopathy (DR)^25,26^ or to determine the severity of age-related macular degeneration (AMD) or DR^27,28^. A more sophisticated network (i.e. Inception-v3) architecture and the EyePACS and Messidor-2 dataset were used to develop and validate the grading of retinal images into normal versus referable DR or referable diabetic macular odema, or both^29^. Other studies, using a similar approach, have also effectively catagorised DR into different grades^30–32^. Freely available online databeses are also generated for the advancement of this field. The freely available STARE database a retrained VGG19 and AlexNet networks were recently used to classify retinal images into ten different classes of pathologies^33^. The efficacy of the freely available DRIVE dataset and a custom CNN and gray-scale thresholding for segmentation of retinal vasculature has also been reported^34^.

The effectiveness of a CNN to detect cardiovascular risk factors from retinal images has recently been previously demonstrated^35^. This study used Google Inceptionv3 neural-network architecture to distinguish patient characteristics from retinal images such as age, gender, hypertension, and smoking status^35^. The last mentioned study has achieved 0.71% (0.70–0.73%) accuracy as measured by the area under the curve (AUC), using not pre-processed fundus photos. Although that study provided ‘attention maps’ to assist with the areas of the training data that were ‘noticed’ by their model, no further conclusion could have been drawn on the potential physiological changes in the ‘noted’ area that had led to CNN’s acquired knowledge.

As the deleterious effects on cardiovascular health in particular are compounded in patients with diabetes, there is a need to develop effective smoking cessation strategies for patients with diabetes who smoke. However in order to test the efficacy of a smoking cessation strategy, one ideally needs an inexpensive and acceptable objective measure of the patients smoking status. In this project we set out to determine whether, using nothing more than the retinal photograph that was obtained when the patient attended for screening, labelled with self-reported smoking status, whether we could build an algorithm capable of detecting whether the individual smoked or not. In this paper, we report the efficacy of our custom-designed CNN for the automated prediction of smoking status, in a self-reported population, using a diabetic retinal screening dataset. Furthermore, by using two pre-processing methods as oppose to common unprocessed fundus photos, we have attempted to create a better understanding of the ‘learned’ knowledge by our CNN and address its ‘black box’ nature.

## Methods

The current clinical study was congruent with the ethical principles conveyed in the 2002 version of the Helsinki Declaration and accepted by the Ethical Committee of New Zealand Health and Disability Ethics Committee, reference #18CEN124. The local regulatory authority in New Zealand (National Ethics Advisory Committee) has waived the need for informed consent. After obtaining ethical approval, 165,104 retinal images were obtained from the Auckland Diabetic Eye Screening Database. All patients in this dataset therefore have diabetes, the grade of the diabetic retinopathy being graded to the New Zealand Ministry of Health Diabetic retinopathy standard^36^. The images had been de-identified and were labelled as “smoking” or “non-smoking” based on the patient’s self-reported smoking status.

The images which were obtained in Auckland Diabetic screening during 2009–2018, were coloured (RGB), in JPG format and resized (320 * 320 pixels) to fit the input criteria of our neural network. Coloured fundus images with one target label: smoking-status (Yes/No) were then split randomly to 60% ‘training set’, 20% ‘validation set’ and 20% ‘test set’. Prior to CNN training, these images were pre-processed using two different filtering methods. Next, the same CNN architecture and hyper-parameters were used for model training (using the ‘training set’) and validation (using the ‘validation set’). Finally, the CNN performance was checked using the not-used-before ‘test set’. The outcome presented in the Results section are from this ‘test set’.

Experiments were run in an Intel Xeon Gold 6128 CPU @ 3.40 GHz with 16 GB of RAM memory and a NVIDIA GeForce TiTan V VOLTA 12 GB, for 20 epochs and training lasted 8 hours. The 20 EPOCHs training-stop criterion was chosen as it was observed that the CNN validation loss (measured as negative log-likelihood and residual sum of squares) has reached a stable minimum over the last 3 EPOCHs of training. Hence, any further training would have led to model ‘over-fitting’, in which the neural network ‘memorizes’ the training examples.

### Pre-processing

**Figure 1:**
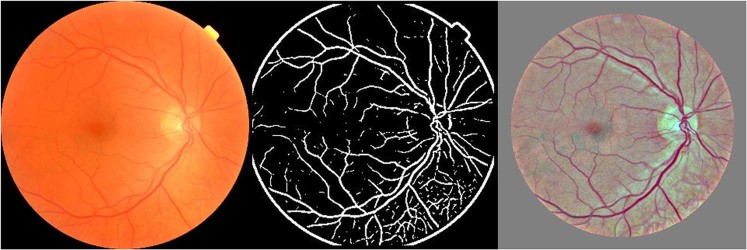
Showing the original (left), skeletonized (centre) and contrast enhanced (right) fundus images.

The images were filtered in two ways (1) “skeletonized”, and (2) “contrast-enhanced” Fig. 1.

The “skeletonization” process was based on a previously published model^37^. Briefly, after loading the fundus image, the green channel of the image was extracted as it provided the highest contrast between the background and the blood vessels. The resultant grey-scale image was then thresholded to improve the contrast of the blood vessels. Luminance was then inverted so that the blood vessels show up as bright pixels against a dark background in grayscale. A Gaussian filter was then applied to smooth the image. For each pixel in the image, the Hessian and Eigen values of the Hessian (λ_1_, λ_2_, where |λ_1_| < |λ_2_|) were computed. The eigenvalues were used to compute measures:

$${R}_{B}=\frac{{\lambda }_{1}}{{\lambda }_{2}}$$

and

$$S=\sqrt{{\lambda }_{1}^{2}+{\lambda }_{2}^{2}}$$

The Vesselness measure for each pixel was then calculated using:

$$V=\{\begin{array}{c}0,\,{\rm{if}}\,{{\rm{\lambda }}}_{2} > 0\\ \exp (-\frac{{R}_{B}^{2}}{2{\beta }^{2}})(1-\exp (-\frac{{S}^{2}}{2{c}^{2}})),\end{array}$$

where *β* and *c* are threshold parameters which control the sensitivity of the Vesselness filter.

The Vesselness measure indicated the probability of a pixel being a vessel. Thresholding was applied and all pixels with a probability higher than the threshold value were assigned as pixels belonging to a vessel Fig. 1B.

“Contrast-enhanced” dataset was obtained using another published method^38,39^. Here, the following Gaussian filter was applied to the original fundus photo:

$${I}_{c}=\alpha I+\beta G(\rho )\,\ast I\,+\gamma $$

where * denotes the convolution operation, *I* denotes input image and *G*(*ρ*) represents the Gaussian filter with a standard deviation of *ρ*^19^ Fig. 1C. These images were then normalized to prevent the well-documented CNN “gradient explosion problem”^40^.

### CNN model

**Figure 2:**
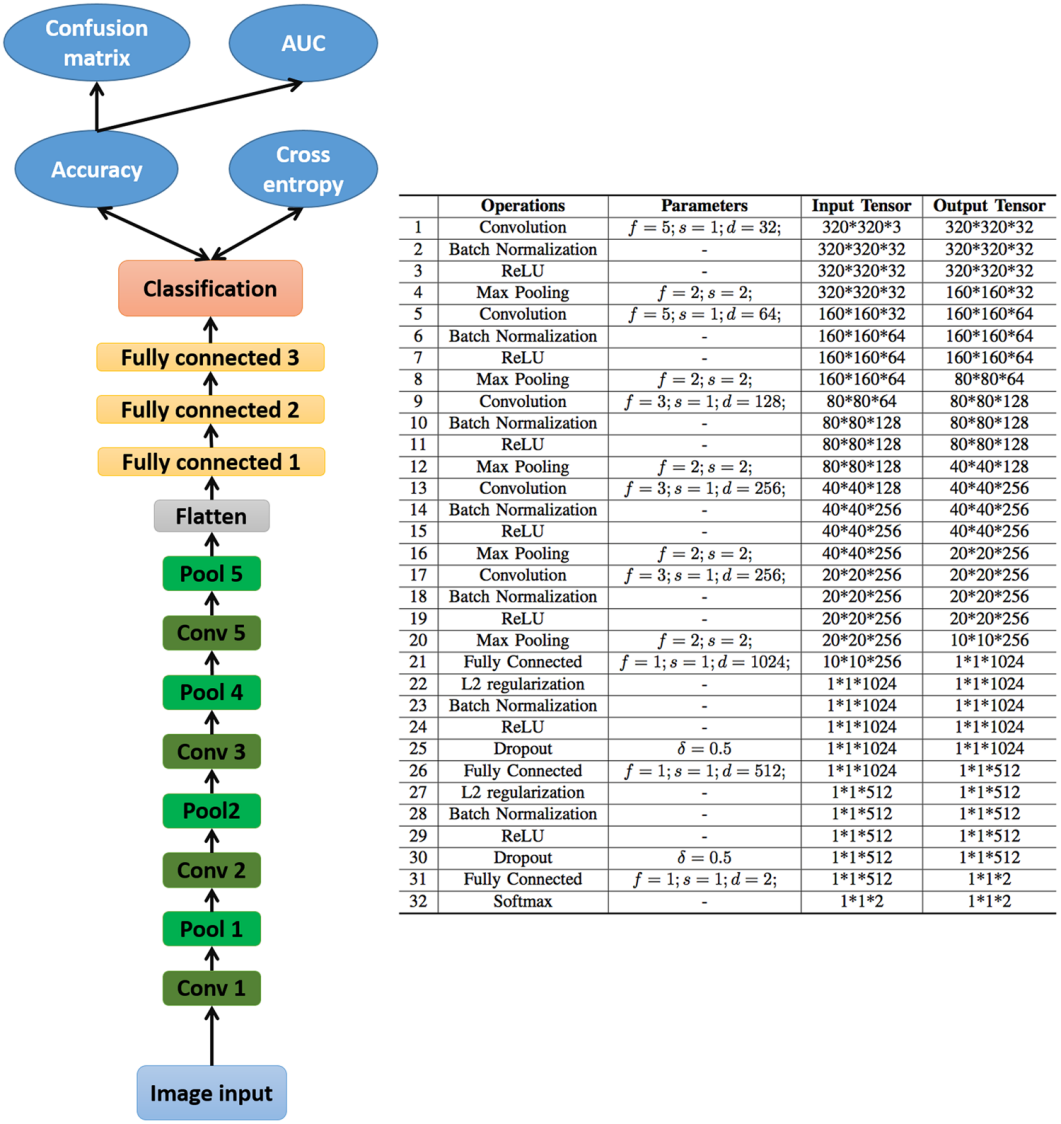
Showing the CNN architecture (left) and each CNN layer property (right).

The CNN architecture that was used in this project is presented below Fig. 2. In short, five convolution layers, five pooling layers and three fully-connected layers composed the main body of our CNN. Batch normalization layers were also added for accelerating converge, dropout and regularization layers were also added to prevent overfitting.

Within the dataset, 85% of the images were self-reported as non-smokers and only 15% as smokers. Imbalanced distribution is common in medical datasets (e.g. healthy vs diseased); and can lead to CNN imbalanced learning^41^. In order to address this issue, the smoker-labelled images were augmented and replicated so that each training mini-batch had similar number of smoker and non-smoker images. This data augmentation strategy was not applied during the validation process.

### Evaluation metrics

For our model evaluation, we adopted several evaluation parameters. These included accuracy, specificity, sensitivity, and area under receiver operating characteristic curve (AUC).

$$Specificity=\frac{TN}{TN+FP}$$

$$Sensitivity=\frac{TP}{TP+FN}$$

$$Accuracy=\frac{TP+TN}{TP+TM+FP+FN}$$

*FP* represents false positive values, where non-smoking images are wrongly classified as smoking and *FN* represents false negative values where smoking images are wrongly classified as non-smoking. *TP* represents correctly classified smoking images and *TN* represents correctly classified non-smoking images.

### Attention maps

To better understand how our better-performing (i.e. contrast-enhanced) CNN, attention maps were produced, to identify the areas on the retinal image that had been used as a predictive feature for smoking status. This method is explained in detail elsewhere^42–44^. Briefly, the output of the first two convolutional layers (layer 1 and layer 5 in Fig. 2) were extracted and averaged for all cases.

## Results

165,104 photographs obtained from 81,711 participants were used in this study. The data was drawn from the Auckland diabetes screening program, and all suitable images from screening visits 2008–2018 inclusive were used. Of the cohort of patients from whom these images were drawn from, 7354 (9%) of them identified as being a current smoker. The demographics of the population, and the status of the diabetic retinopathy from which these fundus photographs were obtained is detailed in Table 1. 66% of the cohort who identified as smokers were male compared to 52% of the cohort in the cohort who identified as non-smokers, a difference that was statistically significant. Regression analysis revealed that there was also a statistically significant relationship between gender and smoking (p < 0.001).

**Table 1:** Demographics of the patients from who the image dataset was derived.

	Non-smoker (74,357 individuals)	Smoker (7354 individuals)
Age mean (SD)	63.6 ± 16.7	58.8 ± 13.2
Gender (%Male)	52%	66%
HbA1c mmol/mol	64 ± 10.4	64 ± 10.7
Dyslipidaemia on treatment.	61%	63%
Hypertension on treatment	64%	62%
Retinopathy level		
R0	46%	45%
R1	35%	36%
R2	15%	14%
R3	2%	3%
R4-5	2%	2%

There was no significant difference in the proportion of patients who were being treated for hypertension or dyslipidaemia in the group who self-identified as smokers compared to those who identified as non-smokers. The diabetic control, as assessed by HbA1C taken closest to the date of the screening event, was well matched between groups. Whilst there was a trend for smokers to be younger, this difference was not statistically significant. The majority of patients in both groups had at least mild non proliferative diabetic retinopathy, but there was no difference in the level of retinopathy between the two groups.

**Figure 3:**
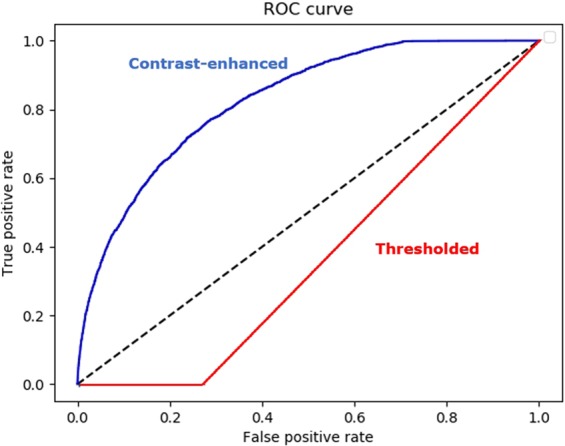
The ROC plots of the contrast-enhanced and thresholded -trained datasets.

Both skeletonized and contrast-enhanced images were used independently, to train and test a CNN model. Using the skeletonized test dataset, our CNN achieved an accuracy of 63.63%, a specificity of 65.60% and a sensitivity of 47.14%. The AUC was 0.58. Using the contrast-enhanced image dataset our CNN produced a superior overall test accuracy 88.88%, and an improved specificity 93.87% and sensitivity of 62.62%. Finally, the AUC was 0.86 Fig. 3.

**Figure 4:**
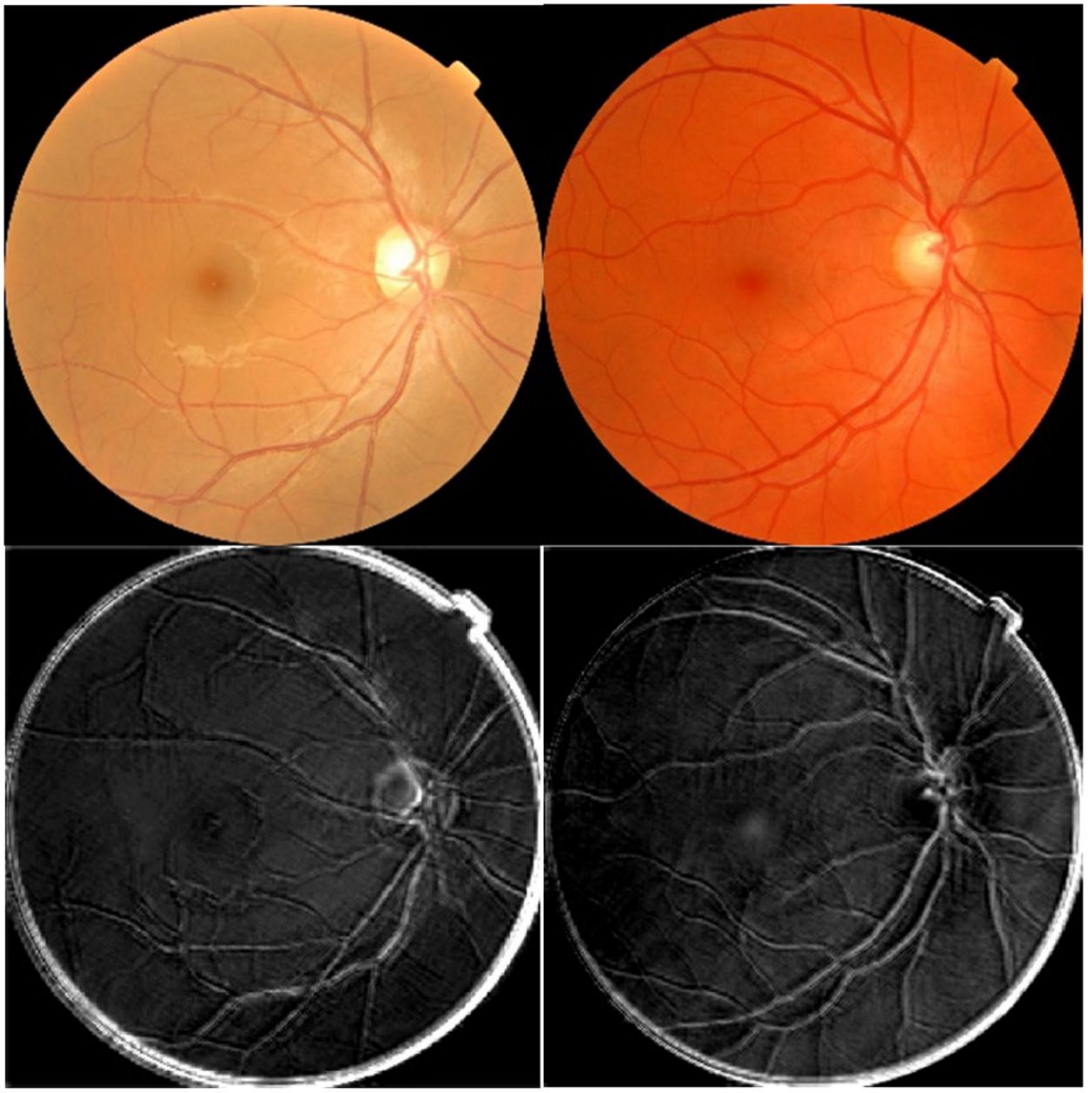
The fundus photos (top row) and attention maps (bottom row) of the enhanced dataset, from a smoker (left) and non-smoker (right) participant, demonstrating the sensitivity of the CNN to the perivascular area.

Representative examples of the attention maps derived from the contrast-enhanced fundus image dataset are shown in Fig. 4. Attention maps were not generated from the skeletonised image dataset as this CNN failed to reach 80% accuracy. Within the attention map the retinal vessels, the perivascular region and the fovea have been highlighted, indicating that the CNN used data from these areas when making its decision on the smoking status of the image under test. The attention maps were similar for all the analysed images and there was no visually identifiable difference between the attention maps derived from the images that were obtained from smokers compared to those from non-smokers.

## Discussion

This study has shown that Computational Neural Networks can be utilised to accurately predict smoking status from retinal fundus images.

In highlighting the retinal vasculature, the perivascular region and the fovea, the attention maps derived from the analysis of the contrast-enhanced image dataset, demonstrate that the CNN has identified these regions on the images as being the most important for predicting the smoking status. A wider retinal venular calibre has previously been reported to be linked to smoking^10,11^, so the finding that the retinal vasculature is highlighted on the attention maps suggests that the CNN is, at least in part, deriving a conclusion based on these structural changes. Similar findings were reported by Poplin, *et al*.^35^, although a lower accuracy 0.71% (0.70–0.73%) as measured by AUC, and using non pre-processed fundus photos. We believe that the main reason why our network outperformed the previous study has been the use of pre-processed fundus photos, as oppose to unprocessed images. We believe that providing our CNN with ‘contrast enhanced’ features of the perivascular region has assisted with its classification task. The observation that removing this region of fundus photos in our ‘skeletonized’ images led to much poorer performance would further support this hypothesis. Meanwhile, it is interesting to observe that a CNN trained on skeletonised images, a pre-processing method, which reduces the image to a geometric representation of vasculature isolated from the rest of the fundus, is unable to accurately classify the smoking status of the images. It suggests that changes in the architecture of the vasculature, such as vessel calibre^6–12^, vessel tortuosity and bifurcation^5^, and vascular fractal dimentions^15–17^ alone are not sufficiently strong predictive markers for the accurate detection of the smoking status of an individual who has diabetes.

Whilst it is difficult to identify with any certainty why the skeletonised model failed to accurately predict whether an individual smoked, this model does not make a clear a distinction between the retinal arteries and veins and it includes some of the larger choroidal vessels Fig. 1. This lack of clarity between the different components of the retinal vasculature and the inclusion of some of the larger choroidal vessels may have introduced noise into model, which reduced its accuracy. Moreover, the finding that the paravascular area and fovea in the contrast-enhanced images are also important indicates that the CNN is deriving important predictive data from these areas. Both hypertension and dyslipidaemia are associated with well recognised changes within the retinal vasculature^45–48^ and it is therefore possible that the paravascular changes that the algorithm used to classify the images were based, at least in part, on these changes. However, the relationship between chronic cigarette smoking and hypertension is inconclusive with large epidemiological studies concluding that any independent chronic effect of smoking on blood pressure is small^49,50^. The one exception perhaps being older male smokers had higher systolic BP adjusted for age, BMI, social class, and alcohol intake compared to matched non-smoking peers^51^. Moreover, nearly two thirds of patients in our study were on treatment for hypertension, and the proportion of patients who were being treated for hypertension was similar between those who identified as smokers, compared to those who identified as non-smokers. These data are likely to reflect that patients with diabetes will have their blood pressure checked regularly as part of their regular systemic review and be treated appropriately. One has to acknowledge that an individual being on treatment for hypertension, is not the same as knowing what their blood pressure is. It is therefore still possible that the cohort who identified as smokers had more signs of hypertensive retinopathy compared to non-smokers. However, as the majority of these individuals were regularly being reviewed by their physician, and that the blood pressure targets for each cohort would be similar, it is probably reasonable to assume that the blood pressure control in both cohorts was also similar. The finding that the HbA1C was very similar in each cohort suggests that the management of their diabetes and associated co-morbidities was similar across groups. Resolving this uncertainty would require the retinal images to be labelled with either the blood pressure at the time of image acquisition, and/or whether hypertensive retinopathy was present. However, this data was not available to us so we were unable to test this hypothesis. The possible influence that dyslipidaemia had on the function of our algorithms can be addressed with a very similar set of arguments, but again we did not have the data, which would allow us to interrogate this association any further.

For any cohort of patients with diabetes one potential confounding variable is the proportion of patients with retinopathy of differing levels. Whilst the majority of patients in this study had at least mild non proliferative diabetic retinopathy, the actual proportions of patients who had retinopathy levels R0-R5 were very similar. Moreover, the attention maps suggest that the algorithms were not sensitive to changes within the retina beyond the larger vessels implying that the level of a patients retinopathy had very little influence on the output of the algorithm.

Whilst CNNs are as a rule fairly insensitive to subtle differences in colours, one has to consider the possibility that the colour, or differences in the colour between different components of the fundal image, could be an important discriminator. The oxygen carriage of haemoglobin is reduced in smokers^52^, something that might affect the colour of the blood in the vessels and the sub foveal choroid. The finding that the fovea was also highlighted in the derived attention maps, albeit to a lesser extent than the retinal vasculature and perivascular region was unexpected. Whilst this finding has previously been considered to be a result of the centrality of the fovea in retinal images^53^, it could also reflect the fact that the CNN was detecting a difference in the colour of the subfoveal choroid in smokers compared to non-smokers. However, in a previous study^35^, the fovea was highlighted on the attention maps that predicted gender. How the CNN was able to accurately predict gender is unknown, but we know that the central macular thickness, as measured by OCT, is thicker in males compared to females, so it is conceivable that the CNN was able to discern this difference^54^. One therefore also has to consider the possibility that there is an inherent bias in our dataset with smoking being unbalanced between females and males and that our CNN is, at least in part, simply reporting this difference. The finding that a greater proportion of our cohort who identified as being a current smoker were male is in keeping with data obtained from the 2013 New Zealand census which found that smoking prevalence was higher in men (16.4%) than women (13.9%)^55^. It is however of note that the prevalence of smoking in our cohort of patients with diabetes who smoked (9%) was actually less than the national average (15%).

It seemed in our data that there was a significant relationship between self-reported smoking and gender. It is possible then that the algorithm was sensitive to gender when making a judgement about the smoking status of an individual, particularly when labelling them a non-smoker. However, the observation that the attention maps were not focused solely on the fovea strongly suggests that the algorithm was not making judgements based on gender alone. Repeating the study with a cohort of smokers and non-smokers who are matched for gender would potentially help address the question as to what influence gender has on the algorithm.

These findings demonstrates both the utility of attention maps to assess which factors the algorithm is using to determine its judgement, but they also highlight the need to be cognisant of the fact that there may be unexpected factors that are powerful confounding variables which may influence the algorithms behaviour. At the extreme, the CNN could even be using a different, but strongly related variable, as a surrogate for that factor you are using the CNN to make a judgement on. This phenomenon probably explains why many apparently well functioning algorithms perform poorly when presented with a dataset derived from a different population, in which these co-variables are inadvertently balanced differently.

Despite the possible anatomical correlations between the predictive features identified on the activation maps and the previously described vascular changes identified in the retinal vasculature of smokers, neither these previous results nor our current data could prove causation. Given the significant number of variables likely to have been analysed by our CNN, and that fact that some of these factors may be unknown to us, in reality we can only speculate on the predictive features used by our CNN for determining smoking status identification in this dataset.

The strengths of this study include the large number of images that were analysed and the use of validated pre-processing image methods. Potential limitations include the imbalanced distribution of smoking and non-smoking images within the dataset and the fact that we were not in a position to balance other cofounding variables, such as gender, that could effected the way the algorithm behaved. This could have led to sampling bias. Data augmentation has been used commonly in DL to address the imbalanced data issue and has also been implemented here. Using this technique, similar number of non-smoking and (augmented) smoking fundus images were included in each min-batch of the CNN training process.

Finally, all the images analysed in the study were taken from a diabetic retinopathy screening database. Patients with diabetics are known to have retinal vascular changes related to the duration and severity of disease^56,57^ and these diseases relate alterations may also have confounded our analysis of the images. However, smoking has previously been found to have one of the largest influences on retinal vessel calibre, independent of other factors in large study evaluating retinal vasculature changes in a diabetic population^58^.

While our CNN has high degree of accuracy and specificity, it suffers from low sensitivity. In other words, the model could distinguish non-smokers from smokers with high degree of accuracy but did not perform as well in identifying smokers. Previous studies have reported that the vascular calibre changes are greatest in those who have the highest number of “pack years”^58^. It is therefore likely that the influence of smoking on the vasculature could be both subtle and cumulative, and as such, these changes may not be apparent until the individual’s smoking habit exceeds a certain threshold. Furthermore, confirmation of smoking status in our study was both self-reported and binary; yes/no. Since our data lacked the frequency, duration, or extent (dose) of an individual smoking behaviour, it is very possible that our CNN was not able to detect with any reliability those individuals whose smoking behaviour was either below a given threshold or were ex-smokers. More information regarding duration and dosage of smoking, including ex-smokers, would have allowed a gradient association pattern between the smoking “dose” and the retinal vasculature to have been analysed.

In summary, we have demonstrated that a CNN analysis of image enhanced retinal photographs can determine smoking status with a high degree of accuracy. Further research is required to improve the accuracy and sensitivity of the model by controlling for more potential confounding variables including ex-smoking status, number of cigarettes smoked and frequency. Further exploration of whether this technique can be used to determine other cardiovascular risk factors from retinal images will require access to data-sets that are gathered from both the general population (i.e. arguably largely healthy individuals) as well as those that are derived from health-care based systems.

## Data Availability

The data that support the findings of this study are available from Auckland Diabetic Eye Screening Database, but restrictions apply to the availability of these data, which were used under license for the current study, and so are not publicly available. Data are however available from the authors upon reasonable request and with permission of Auckland Diabetic Eye Screening Database.
